# Association among weight change, glycemic control, and markers of cardiovascular risk with exenatide once weekly: a pooled analysis of patients with type 2 diabetes

**DOI:** 10.1186/s12933-014-0171-2

**Published:** 2015-02-03

**Authors:** Lawrence Blonde, Richard Pencek, Leigh MacConell

**Affiliations:** Department of Endocrinology, Ochsner Medical Center, 1514 Jefferson Highway, 70121 New Orleans, LA USA; Bristol-Myers Squibb/AstraZeneca, San Diego, CA USA

**Keywords:** Exenatide, Type 2 diabetes mellitus, Hyperglycemia, Weight response, Cardiovascular risk, Biomarkers

## Abstract

**Background:**

Overweight or obesity contributes to the development of type 2 diabetes mellitus (T2DM) and increases cardiovascular risk. Exenatide, a glucagon-like peptide-1 receptor agonist, significantly reduces glycated hemoglobin (A1C) and body weight and improves cardiovascular risk markers in patients with T2DM. As weight loss alone has been shown to reduce A1C and cardiovascular risk markers, this analysis explored whether weight loss contributed importantly to clinical responses to exenatide once weekly.

**Methods:**

A pooled analysis from eight studies of exenatide once weekly was conducted. Patients were distributed into quartiles from greatest weight loss (Quartile 1) to least loss or gain (Quartile 4). Parameters evaluated for each quartile included A1C, fasting plasma glucose (FPG), blood pressure (BP), heart rate, high-density lipoprotein cholesterol (HDL-C), low-density lipoprotein cholesterol (LDL-C), total cholesterol, triglycerides, and the liver enzymes alanine aminotransferase (ALT) and aspartate aminotransferase (AST).

**Results:**

The median changes from baseline in body weight in Quartiles 1–4 were −6.0, –3.0, −1.0, and +1.0 kg, respectively. All quartiles had reductions in A1C (median changes −1.6, −1.4, −1.1, and −1.2%, respectively) and FPG (−41, −40, −31, and −25 mg/dL, respectively), with the greatest decreases in Quartiles 1 and 2. Most cardiovascular risk markers (except diastolic BP) and liver enzymes improved in Quartiles 1 through 3 and were relatively unchanged in Quartile 4. Higher rates of gastrointestinal adverse events and hypoglycemia were observed in Quartile 1 compared with Quartiles 2 through 4.

**Conclusions:**

Exenatide once weekly improved glycemic parameters independent of weight change, although the magnitude of improvement increased with increasing weight loss. The greatest trend of improvement in glycemic parameters, cardiovascular risk factors including systolic BP, LDL-C, total cholesterol, and triglycerides, and in liver enzymes, was seen in the patient quartiles with the greatest reductions in body weight.

## Introduction

The increased incidence of new diabetes is significantly associated with the increased incidence and prevalence of overweight and obesity [1-3] and reduction of excess body weight may be helpful for patients with diabetes. Treatment guidelines recommend weight loss and greater physical activity as part of a strategy to reduce risk of progression from prediabetes to overt type 2 diabetes mellitus (T2DM) [4,5]. It is also a corner stone of treatment for those with type 2 diabetes and a complement to glucose-lowering pharmacotherapy [4-6]. Weight reduction alone may improve glycemic control and have beneficial effects on cardiovascular (CV) risk factors. In the Look AHEAD (Action for Health in Diabetes) study, patients randomized to intensive lifestyle interventions lost 8.6% of their body weight from baseline after 1 year, and this weight reduction was accompanied by decreases in glycated hemoglobin (A1C) (−0.6%) and fasting plasma glucose (FPG) (−21.5 mg/dL) [7]. Multiple CV risk factors were also ameliorated, though the 5% decrease in the primary end point, a composite of cardiovascular death, nonfatal myocardial infarction or stroke, or hospitalization for angina, was not statistically significant (P = 0.51) [7,8].

Exenatide, a glucagon-like peptide-1 receptor agonist (GLP-1RA), which is available in twice daily or once weekly formulations, has been shown to improve glycemic control and reduce excess body weight in patients with T2DM. Studies of exenatide once weekly demonstrated mean reductions in A1C ranging from −1.3% to −1.9% and mean weight reduction ranging from −2.0 kg to −3.7 kg [9-16]. Exenatide once weekly has also been associated with the significant improvement of a variety of CV risk markers, including blood pressure, lipids, and anthropomorphic measurements [9,11-16].

The contribution of weight loss to the clinical effects observed with exenatide once weekly is not known. An analysis was conducted to determine if improvements in glycemic parameters and CV risk markers might result from or occur independently of body weight loss.

## Materials and methods

Pooled data were analyzed using the intent-to-treat (ITT) patient population receiving exenatide once weekly, with or without oral glucose-lowering medications, from eight randomized, controlled 24- to 30-week trials (Table 1) [9-16]. Patients enrolled in the studies were at least 16 years of age with T2DM, an A1C of 7.1 to 11.0%, stable body weight (3–6 months prior to enrollment), and body mass index (BMI) of 23 to 45 kg/m^2^. Quartiles were created by dividing the total patient population into four approximately equal subgroups based on body weight change from baseline: Quartile 1 consisted of the 25% of subjects with the greatest weight loss at the end of the controlled period; Quartile 4 consisted of the 25% of subjects with the smallest weight reduction (or weight gain).

**Table 1:** **Characteristics of exenatide once weekly studies**

**Author**	**N**	**Study duration (weeks)**	**Baseline A1C (%)** ^**a**^	**Change in A1C (%)** ^**a,b**^	**Baseline body weight (kg)** ^**a**^	**Change in body weight (kg)** ^**a,b**^
Drucker et al. 2008 [14]	148	30	8.3	−1.9	102	−3.7
(DURATION-1)						
Bergenstal et al. 2010 [9]	160	26	8.6	−1.5	89	−2.3
(DURATION-2)						
Diamant et al. 2010 [13]	233	26	8.3	−1.5	91.2	−2.6
(DURATION-3)						
Russell-Jones et al. 2012 [16]	248	26	8.5	−1.5	87.5	−2.0
(DURATION-4)						
Blevins et al. 2011 [10]	129	24	8.5	−1.6	97.0	−2.3
(DURATION-5)						
Buse et al. 2013 [11]	461	26	8.5	−1.3	90.9	−2.7
(DURATION-6)						
Davies et al. 2013 [12]	111	26	8.4	−1.3	96.7	−2.7
Ji et al. 2013 [15]	340	26	8.7	−1.4	69.6	−1.6

A1C: glycated hemoglobin; ^a^All mean or least-squares mean values; ^b^At study end point.

Laboratory data available for each study included A1C, FPG, high-density lipoprotein cholesterol (HDL-C), low-density lipoprotein cholesterol (LDL-C), total cholesterol, triglycerides, alanine aminotransferase (ALT), and aspartate aminotransferase (AST). A single laboratory was used for the measurements in each study, but the same laboratory was not used for all studies. Vital signs available for each study included systolic blood pressure (SBP), diastolic blood pressure (DBP), and heart rate (HR). Laboratory data and vital signs were collected at baseline and at regular intervals through study end point.

In the analysis, efficacy data included glycemic parameters (A1C and FPG) and CV risk markers (SBP, DBP, HR, HDL-C, LDL-C, total cholesterol, and triglycerides), and safety data included liver enzymes (ALT and AST), the number of adverse events, and the incidence of severe and non-severe hypoglycemia. Severe hypoglycemia was defined as symptoms resulting in loss of consciousness or seizure that showed prompt recovery after administration of glucose, or documented blood glucose <54 mg/dL that required third-party assistance because of severe impairment in consciousness or behavior. Non-severe hypoglycemia was defined as signs or symptoms of hypoglycemia accompanied by fingerstick blood glucose <54 mg/dL. Baseline demographics and adverse events were summarized by descriptive statistics by weight quartile. Efficacy data and liver enzymes were evaluated using last observation carried forward (LOCF) changes from baseline to end point with median changes by quartile and mean changes with 95% confidence intervals (CIs) calculated by quartile and for the ITT population. To examine the linear dependence of the change in body weight on the change in A1C, Pearson correlation coefficients were calculated by quartile and for the ITT population.

Studies included in the analysis were conducted in accordance with the Declaration of Helsinki. Study protocols were approved by an institutional review board at each investigator site and all patients provided written informed consent.

## Results

**Figure 1:**
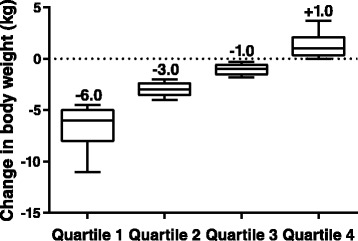
**Distribution of median change in body weight, by end-point body weight change quartiles.** The central line represents the median (also labelled above the plot), the box encloses the 25th to 75th percentiles of the distribution, and the outer bars are drawn to the 10th and 90th percentiles.

The analysis included pooled data from 1830 patients. Patient demographic and clinical characteristics by quartile and for the population as a whole are shown in Table 2. Patients had at baseline a mean age of 55 years, mean BMI 31.5 kg/m^2^, mean A1C 8.5%, mean BP 130.8/79.1 mm Hg, and mean LDL-C 100.4 mg/dL. At baseline, the majority of patients were taking metformin (79.4%), less than half were taking a sulfonylurea (43.6%), and less than one-fifth were treated only with lifestyle interventions of medical nutrition therapy and physical activity (15.9%). Baseline characteristics in different quartiles of weight loss were mostly evenly distributed with respect to age and known diabetes duration. Mean A1C and FPG baseline values were the lowest in Quartile 1 and trended upward across body weight change quartiles. Body weight was reduced in three of four quartiles. The median change from baseline in body weight was −6.0 kg in Quartile 1, −3.0 kg in Quartile 2, −1.0 kg in Quartile 3, and +1.0 kg in Quartile 4 (Figure 1).

**Table 2:** **Patient demographics and clinical characteristics**

**Characteristic**	**Quartile 1 (−37.7 to −4.1 kg)** ^**a**^ **(n = 455)**	**Quartile 2 (−4.1 to −2.0 kg)** ^**a**^ **(n = 458)**	**Quartile 3 (−2.0 to −0.1 kg)** ^**a**^ **(n = 450)**	**Quartile 4 (−0.1 to +21.3 kg)** ^**a**^ **(n = 455)**	**Total (N = 1830)**
Male, n (%)	223 (49.0)	249 (54.4)	257 (57.1)	283 (62.2)	1015 (55.5)
Age (y), mean (SD)	56 (9)	57 (10)	55 (10)	54 (11)	55 (10)
Known diabetes duration (y), mean (SD)	7 (6)	7 (6)	7 (6)	6 (5)	7 (6)
Race, n (%)					
White	320 (70.3)	284 (62.0)	245 (54.4)	251 (55.2)	1104 (60.3)
Black	8 (1.8)	9 (2.0)	14 (3.1)	16 (3.5)	48 (2.6)
Hispanic	32 (7.0)	36 (7.9)	34 (7.6)	42 (9.2)	147 (8.0)
Asian	89 (19.6)	123 (26.9)	153 (34.0)	142 (31.2)	511 (27.9)
Other	6 (1.3)	6 (2.6)	4 (0.9)	4 (0.9)	20 (1.1)
Glucose-lowering background treatment, n (%)^b^					
Diet and exercise only	72 (15.8)	52 (11.4)	72 (16.0)	90 (19.8)	291 (15.9)
Insulin	3 (0.7)	1 (0.2)	3 (0.7)	1 (0.2)	8 (0.4)
Metformin	373 (82.0)	388 (84.7)	355 (78.9)	330 (72.5)	1453 (79.4)
Thiazolidinedione	22 (4.8)	19 (4.1)	27 (6.0)	32 (7.0)	100 (5.5)
Sulfonylurea	172 (37.8)	212 (46.3)	207 (46.0)	201 (44.2)	798 (43.6)
A1C (%), mean (SD)	8.3 (1.0)	8.4 (1.0)	8.5 (1.1)	8.7 (1.1)	8.5 (1.1)
FPG (mg/dL), mean (SD)	167.0 (44.2)	170.9 (45.7)	170.9 (46.2)	175.6 (50.8)	171.2 (46.9)
Body weight (kg), mean (SD)	93.7 (21.0)	85.3 (18.9)	85.3 (19.1)	87.7 (21.3)	88.0 (20.4)
BMI (kg/m^2^)					
Mean (SD)	33.3 (5.7)	30.8 (5.6)	30.8 (5.3)	30.9 (5.6)	31.5 (5.6)
Category					
<30	143 (31.4)	222 (48.5)	216 (48.0)	217 (47.7)	801 (43.8)
≥30	310 (68.1)	235 (51.3)	228 (50.7)	236 (51.9)	1018 (55.6)
Missing	2 (0.4)	1 (0.2)	6 (1.3)	2 (0.4)	11 (0.6)
SBP (mm Hg), mean (SD)	130.5 (14.8)	131.6 (15.2)	131.3 (14.3)	129.9 (14.2)	130.8 (14.7)
DBP (mm Hg), mean (SD)	78.6 (8.8)	78.7 (9.2)	80.1 (8.6)	79.1 (9.2)	79.1 (8.9)
HR (beats per minute), mean (SD)	74.1 (9.4)	74.8 (9.1)	75.2 (9.6)	74.9 (9.8)	74.7 (9.5)
ALT (U/L), mean (SD)^c^	32.8 (19.2)	32.2 (19.1)	33.4 (19.6)	34.2 (21.8)	33.1 (19.9)
AST (U/L), mean (SD)^d^	26.9 (12.8)	25.9 (12.7)	26.2 (11.2)	26.4 (12.2)	26.3 (12.2)
Lipids (mg/dL), mean (SD)					
Total cholesterol	176.7 (42.3)	178.3 (43.4)	177.2 (41.7)	184.9 (43.2)	179.3 (42.7)
LDL-C	98.7 (34.7)	99.5 (37.2)	99.2 (34.5)	104.3 (37.9)	100.4 (36.0)
HDL-C	43.9 (11.85)	44.2 (11.54)	44.9 (12.32)	45.2 (12.52)	44.5 (12.1)
Triglycerides	183.0 (147.1)	184.8 (147.0)	175.5 (121.6)	190.5 (141.4)	183.5 (139.6)

A1C: glycated hemoglobin; ALT: alanine aminotransferase; AST: aspartate aminotransferase; BMI: body mass index; DBP: diastolic blood pressure; FPG: fasting plasma glucose; HDL-C: high-density lipoprotein cholesterol; HR: heart rate; LDL-C: low-density lipoprotein cholesterol; SBP: systolic blood pressure; SD: standard deviation; ^a^Range of weight change per quartile; ^b^Values are presented as incidence of use, with or without other glucose-lowering therapies, unless otherwise indicated; ^c^ALT normal values: 10 to 40 U/L; ^d^AST normal values: 10 to 34 U/L.

Some inter-quartile differences in patient baseline characteristics were noted (Table 2). The proportion of male patients was greatest in Quartile 4, with the smallest proportion in Quartile 1. Metformin use was the highest in Quartile 2 and the lowest in Quartile 4, and sulfonylurea use was the highest in Quartile 2 and the lowest in Quartile 1. Baseline body weight was the highest in Quartile 1 compared with the remaining quartiles, which were mostly similar, and the highest baseline lipid values occurred in Quartile 4.

**Figure 2:**
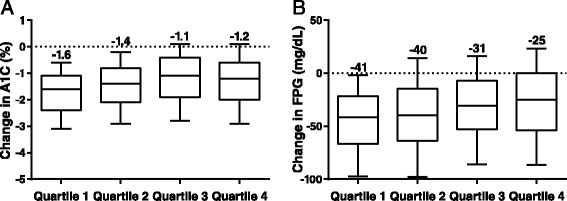
**Median changes in A1C and FPG, by end-point body weight change quartiles. (A)** A1C. **(B)** FPG. A1C, hemoglobin A1C; FPG, fasting plasma glucose. The central line represents the median (also labelled above the plot), the box encloses the 25th to 75th percentiles of the distribution, and the outer bars are drawn to the 10th and 90th percentiles.

Clinically relevant A1C and FPG reductions were observed across all body weight change quartiles. The median change from baseline in A1C was −1.6% in Quartile 1, −1.4% in Quartile 2, −1.1% in Quartile 3, and −1.2% in Quartile 4 (Figure 2A). The mean (standard error) changes in A1C with 95% confidence intervals by Quartile are presented in Table 3.

**Table 3:** **Mean changes in parameters from baseline to end point**

**Parameter, mean (SE) [95% CI]**	**Quartile 1**	**Quartile 2**	**Quartile 3**	**Quartile 4**	**All Patients**
	**(n = 455)**	**(n = 458)**	**(n = 450)**	**(n = 455)**	**(N = 1830)**
Weight (kg)	−7.0 (0.15)	−3.0 (0.03)	−1.0 (0.03)	1.5 (0.09)	−2.4 (0.09)
	[−7.3, −6.7]	[−3.0, −2.9]	[−1.1, −1.0]	[1.4, 1.7]	[−2.5, −2.2]
A1C (%)	−1.7 (0.05)	−1.4 (0.05)	−1.2 (0.06)	−1.3 (0.05)	−1.4 (0.03)
	[−1.8, −1.6]	[−1.5, −1.3]	[−1.3, −1.1]	[−1.4, −1.2]	[−1.5, −1.3]
FPG (mg/dL)	−43.5 (2.30)	−38.3 (2.29)	−31.7 (2.13)	−29.1 (2.50)	−35.7 (1.16)
	[−48.0, −39.0]	[−42.8, −33.8]	[−35.9, −27.6]	[−34.1, −24.2]	[−38.0, −33.5]
SBP (mm Hg)	−5.8 (0.71)	−3.7 (0.68)	−3.1 (0.60)	−0.5 (0.60)	−3.3 (0.33)
	[−7.2, −4.4]	[−5.1, −2.4]	[−4.2, −1.9]	[−1.7, 0.7]	[−3.9, −2.6]
DBP (mm Hg)	−1.1 (0.43)	−1.4 (0.41)	−1.1 (0.40)	0.3 (0.44)	−0.8 (0.21)
	[−1.9, −0.2]	[−2.2, −0.6]	[−1.9, −0.3]	[−0.5, 1.2]	[−1.2, −0.4]
HR (beats per minute)	3.4 (0.48)	2.8 (0.44)	2.2 (0.46)	2.9 (0.48)	2.8 (0.23)
	[2.5, 4.4]	[1.9, 3.7]	[1.3, 3.1]	[1.9, 3.8]	[2.4, 3.3]
HDL-C (mg/dL)	0.9 (0.31)	0.7 (0.30)	0.0 (0.33)	−0.2 (0.34)	0.4 (0.16)
	[0.3, 1.5]	[0.2, 1.3]	[−0.6, 0.7]	[−0.9, 0.5]	[0.1, 0.7]
LDL-C (mg/dL)	−6.1 (1.23)	−5.9 (1.15)	−1.3 (1.25)	−1.0 (1.36)	−3.6 (0.63)
	[−8.5, −3.6]	[−8.2, −3.7]	[−3.8, 1.1]	[−3.7, 1.7]	[−4.8, −2.4]
Total cholesterol (mg/dL)	−9.3 (1.53)	−8.2 (1.39)	−2.5 (1.47)	−1.5 (1.62)	−5.4 (0.76)
	[−12.3, −6.3]	[−11.0, −5.5]	[−5.4, 0.4]	[−4.7, 1.7]	[−6.9, −3.9]
Triglycerides (mg/dL)	−26.1 (7.40)	−19.7 (5.70)	−6.2 (4.64)	0.5 (5.99)	−13.0 (3.02)
	[−40.7, −11.6]	[−30.9, −8.5]	[−15.3, 2.9]	[−11.3, 12.3]	[−19.0, −7.1]
ALT (U/L)	−8.2 (0.67)	−3.3 (0.78)	−2.9 (0.76)	1.2 (1.44)	−3.3 (0.48)
	[−9.6, −6.9]	[−4.8, −1.8]	[−4.4, −1.4]	[−1.7, 4.0]	[−4.3, −2.4]
AST (U/L)	−5.9 (0.77)	−3.3 (0.82)	−1.2 (0.83)	−1.5 (1.06)	−3.2 (0.44)
	[−7.5, −4.4]	[−4.9, −1.7]	[−2.8, 0.5]	[−3.6, 0.6]	[−4.1, −2.3]

A1C: glycated hemoglobin; ALT: alanine aminotransferase; AST: aspartate aminotransferase; CI: confidence interval; DBP: diastolic blood pressure; FPG: fasting plasma glucose; HDL-C: high-density lipoprotein cholesterol; HR: heart rate; LDL-C: low-density lipoprotein cholesterol; SBP: systolic blood pressure.

**Figure 3:**
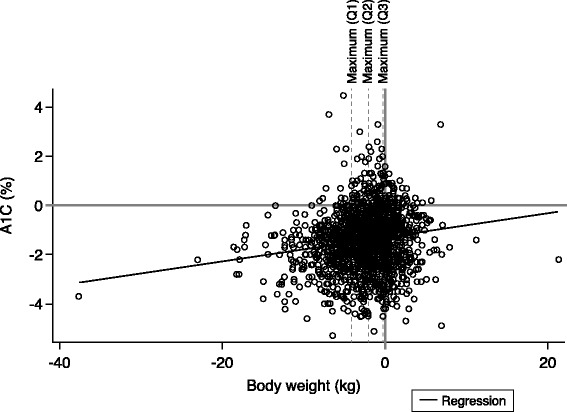
**Pearson linear correlation of change in body weight and change in A1C.** The solid black line represents the regression. Dotted lines represent the maximum body weight change in Quartiles 1 through 3. The r-value for all patients was 0.1579, and r-values for Quartiles 1 through 4 were 0.1847, 0.0267, 0.0485, and −0.0542, respectively. A1C, hemoglobin A1C; Q, quartile.

The mean changes in A1C for all quartiles are presented in Table 3. A higher proportion of patients achieved A1C targets in the quartiles with the greatest weight loss. In Quartiles 1, 2, 3, and 4, an A1C of <7% was achieved by 76.0%, 63.3%, 46.0%, and 37.1% of patients, respectively, and an A1C of ≤6.5% was achieved by 57.6%, 42.1%, 26.9%, and 19.6% of patients, respectively. The scatter-plot along with the regression line exhibits the linear dependence between change in body weight and change in A1C, which was found to be weak overall (Figure 3). The change in median FPG followed a similar pattern across body weight loss quartiles as A1C values: −41 mg/dL in Quartile 1, −40 mg/dL in Quartile 2, −31 mg/dL in Quartile 3, and −25 mg/dL in Quartile 4 (Figure 2B).

**Figure 4:**
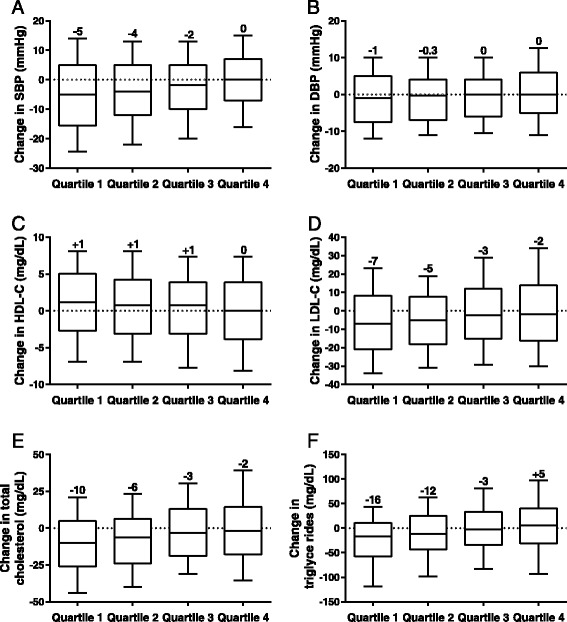
**Median changes in blood pressure and lipids, by end-point body weight change quartiles. (A)** SBP. **(B)** DBP. **(C)** HDL-C. **(D)** LDL-C. **(E)** Total cholesterol. **(F)** Triglycerides. DBP, diastolic blood pressure; HDL-C, high-density lipoprotein cholesterol; LDL-C, low-density lipoprotein cholesterol; SBP, systolic blood pressure. The central line represents the median (also labelled above the plot), the box encloses the 25th to 75th percentiles of the distribution, and the outer bars are drawn to the 10th and 90th percentiles.

The greatest improvements in CV risk markers were observed in Quartile 1, where 50% to 75% of patients showed decreases from baseline in median SBP, LDL-C, total cholesterol, and triglycerides by study end point; HDL-C was slightly increased (Figures 4A–F). In Quartiles 2 and 3, improvements in CV risk markers were smaller compared with those in Quartile 1. In Quartile 4, all CV risk markers were mostly unchanged from baseline except for LDL-C and total cholesterol. The greatest increase in HR occurred in Quartile 1 (mean 3.4 beats per minute) and varied across Quartiles 2 through 4 (mean 2.2–2.9 beats per minute).

**Figure 5:**
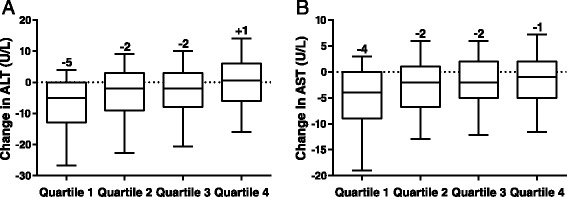
**Median changes in ALT and AST, by end-point body weight change quartiles. (A)** ALT. **(B)** AST. ALT, alanine aminotransferase; AST, aspartate aminotransferase. The central line represents the median (also labelled above the plot), the box encloses the 25th to 75th percentiles of the distribution, and the outer bars are drawn to the 10th and 90th percentiles.

Modest decreases in ALT and AST were observed in the quartile with the greatest body weight reduction (Figures 5A and B). Improvements were minimal or absent across the remaining quartiles. ALT and AST values were within normal limits at study end point.

Treatment-emergent adverse events reported by at least 5% of patients treated with exenatide once weekly in the overall population are shown in Table 4. The most commonly reported adverse events were hypoglycemia (17.3% of patients), nausea (16.7%), and diarrhea (11.3%). Hypoglycemic events were more frequently reported among patients receiving background sulfonylurea therapy compared with those patients who did not receive sulfonylurea treatment (28.8% vs 8.3%).

**Table 4:** **Treatment-emergent adverse events reported by at least 5% of patients treated with exenatide once weekly**

**Adverse event, n (%)**		**Overall (N = 1830)**	
Nausea		305 (16.7)	
Diarrhea		207 (11.3)	
Nasopharyngitis		152 (8.3)	
Headache		145 (7.9)	
Injection site nodule		141 (7.7)	
Vomiting		128 (7.0)	
Constipation		109 (6.0)	
Injection site pruritus		101 (5.5)	
	All*	**Severe** ^**†**^	**Non-severe** ^**‡**^
Hypoglycemia	317 (17.3)		
Background sulfonylurea use	230 (28.8)	0 (0.0)	104 (13.0)
No background sulfonylurea use	86 (8.3)	1 (0.1)	23 (2.2)

*All hypoglycemia: includes severe, non-severe, and symptoms of hypoglycemia (defined as any episode of hypoglycemia that does not meet severe or non-severe event criteria); ^†^Severe hypoglycemia: symptoms resulting in loss of consciousness or seizure that showed prompt recovery after administration of glucose, or documented blood glucose <54 mg/dL that required third-party assistance because of severe impairment in consciousness or behavior; ^‡^Non-severe hypoglycemia: signs or symptoms of hypoglycemia accompanied by fingerstick blood glucose <54 mg/dL.

The incidence of treatment-emergent adverse events was higher overall in the quartile with the greatest weight loss from baseline. The proportion of patients with hypoglycemia decreased across body weight loss quartiles from 20.9% in Quartile 1 to 19.0% in Quartile 2, 16.7% in Quartile 3, and 13.0% in Quartile 4. In each quartile, hypoglycemia rates were higher in sulfonylurea users compared with non-users (Quartile 1: 37.2% vs 11.0%; Quartile 2: 34.0% vs 6.1%; Quartile 3: 25.1% vs 9.1%; Quartile 4: 20.9% vs 6.7%). Gastrointestinal adverse events showed a similar trend with higher rates in Quartiles 1 and 2. Nausea was reported in 22.0% of patients in Quartile 1, 17.9% in Quartile 2, 13.8% in Quartile 3, and 13.2% in Quartile 4.

## Discussion

In this analysis of pooled data, the majority of patients with T2DM treated with exenatide once weekly showed body weight loss. However, clinically meaningful glycemic improvement was seen regardless of change in body weight. Reductions in median A1C and FPG were seen across all quartiles, including Quartile 4, which had modest weight gain, although the correlation between change in weight and change in A1C was found to be weak overall. This suggests that both A1C and weight reduction are indications of a positive response to exenatide; those patients with the most positive response have the greatest reductions in both A1C and weight. The findings of the present analysis are similar to those of two previous analyses of exenatide twice daily and at least one analysis of liraglutide, in which A1C reductions were observed in all body weight change quartiles and the magnitude of glycemic improvement generally was larger in those with greater body weight loss [17-19].

### Effects beyond glycemic control

Beyond the control of glucose and improvement of weight, multiple mechanistic studies in animals suggest that GLP-1 receptor agonism has additional positive effects including increased pancreatic beta cell mass [20], aortic vasodilation [21], cardiac protection against ischemia or reperfusion injury [22], and protection against hepatic lipid accumulation [23]. While the relevance of these findings in humans is unknown, improvements in CV risk markers (excluding DBP) and liver enzymes from baseline were observed with exenatide once weekly in the majority of body weight loss quartiles and with the greatest improvement in the quartile with the greatest reduction of body weight. The magnitude of improvement for these parameters increased with greater weight loss, similar to what was observed in previous analyses of exenatide twice daily by weight loss quartile [17,18,24]. The lack of reductions in DBP across quartiles is in contrast to results observed with one previous analysis of exenatide twice daily that demonstrated a positive relationship between weight loss and improvement in DBP [17]. The present analysis is the first to report changes in HR across body weight loss quartiles with GLP-1RA therapy. The magnitude of HR increase was small overall and the increase became smaller with less body weight loss.

Whether the observed improvements in CV risk markers with exenatide once weekly translate to improvements in CV-related end points or outcomes is not presently known. At least one outcomes study looking into CV end points with exenatide once weekly is currently in progress (Exenatide Study of Cardiovascular Event Lowering Trial [EXSCEL], NCT01144338) and is estimated to be completed in March 2018.

A trend of increased incidence of both gastrointestinal adverse events and hypoglycemia was observed with increased weight loss, the reason for which is not definitively known. Approximately 80% of patients in Quartile 1 lost weight without any nausea events. However, more patients in the highest quartile of body weight loss experienced nausea than in the lowest quartile, suggesting that nausea may be associated with greater weight loss or responsiveness to therapy [25]. At baseline, the majority of patients were taking metformin; thus, it is possible that metformin use may have contributed to the increased incidence of GI adverse events. However, such an effect would be expected to impact all quartiles since baseline metformin use was high across each quartile. The increased incidence of hypoglycemia in patients with increased weight loss might also signal a greater responsiveness (e.g., more insulin secretion) to exenatide therapy. Sulfonylureas have been shown to uncouple the glucose dependence of GLP-1 receptor activation and increase overall insulin secretion in the rat pancreas when co-infused with GLP-1 [26]. Rates of hypoglycemia were higher overall with concomitant sulfonylurea use compared with non-use and followed the same overall trend of increased hypoglycemia with increased weight loss. It should be noted, that the reported rates of hypoglycemia included patients with reported hypoglycemia symptoms not confirmed by fingerstick glucose in addition to the more strictly defined severe and non-severe hypoglycemia; thus hypoglycemia rates may have been higher overall than expected.

### Limitations

Several confounding factors may have impacted the observed results. There were a few imbalances in the baseline demographics across quartiles that could have influenced these findings. For example, glycemic response and use of background sulfonylureas differed among the quartiles, potentially affecting rates of hypoglycemia. Sulfonylurea treatment has also been associated with increases in body weight [27,28]. In addition, patients in Quartile 1 had higher baseline weight and overall obesity, whereas the baseline weight of patients in the other 3 quartiles was more evenly balanced. Finally, patients in Quartile 1 had the lowest A1C at baseline compared with Quartiles 2 through 4. It is possible that this imbalance in baseline A1C combined with the greatest change in body weight and A1C observed in Quartile 1 may have confounded the result of higher A1C goal achievement. Exenatide therapy paired with lifestyle changes may lead to improved clinical efficacy overall. In a study of overweight or obese patients with T2DM who failed to achieve glycemic targets on metformin or sulfonylurea monotherapy, patients randomized to exenatide twice daily plus a program of reduced caloric intake and increased physical activity saw significantly greater reductions in weight (−6.2 vs −4.0 kg), A1C (−1.2% vs −0.7%), SBP (−9.4 vs −2.0 mm Hg), and DBP (−2.2 vs +0.5 mm Hg) compared with those randomized to lifestyle modifications and maintenance on oral therapy [29].

### Implications

The present analysis is consistent with the recommendations of clinical practice guidelines that weight loss, through medical nutrition therapy and appropriately prescribed physical activity, should be a cornerstone of therapy for patients with T2DM [5,6]. However, most patients will also require pharmacologic antihyperglycemic therapy. Improvements in glycemic parameters, most CV risk markers, and liver enzymes with exenatide once weekly were greater in those with the greatest weight reduction. The results of the analysis also support the role of exenatide once weekly as an effective and well-tolerated treatment option for patients with T2DM, the majority of whom are overweight or obese.

## Conclusions

Clinically important reductions in A1C and FPG were seen with exenatide once weekly across all body weight change quartiles, even in those patients with small reductions in body weight or small weight gain, demonstrating that exenatide improved glycemic control independent of weight loss. Nevertheless, the majority of patients achieved reductions in body weight. While all body weight change quartiles showed improvement in glycemic parameters and the majority of quartiles showed improvement in CV risk factors and liver enzymes, the greatest trend of improvement in the efficacy of exenatide once weekly was seen in the patient quartiles with the greatest reductions in body weight.

## Abbreviations

A1C: Glycated hemoglobin
ALT: Alanine aminotransferase
AST: Aspartate aminotransferase
BMI: Body mass index
BP: Blood pressure
CV: Cardiovascular
CI: Confidence intervals
DBP: Diastolic blood pressure
FPG: Fasting plasma glucose
GLP-1RA: Glucagon-like peptide-1 receptor agonist
HDL-C: High-density lipoprotein cholesterol
HR: Heart rate
ITT: Intent-to-treat
LDL-C: Low-density lipoprotein cholesterol
LOCF: Last observation carried forward
SBP: Systolic blood pressure
T2DM: Type 2 diabetes mellitus
